# An Insight into Molecular Targets of Breast Cancer Brain Metastasis

**DOI:** 10.3390/ijms231911687

**Published:** 2022-10-02

**Authors:** Mohammed Kaleem, Mahmood Hassan Dalhat, Lubna Azmi, Turky Omar Asar, Wasim Ahmad, Maimonah Alghanmi, Amal Almostadi, Torki A. Zughaibi, Shams Tabrez

**Affiliations:** 1 Department of Pharmacology, Faculty of Pharmacy, Dadasaheb Balpande College of Pharmacy, Nagpur 440037, India; 2 Department of Biochemistry, Faculty of Science, King Abdulaziz University, Jeddah 21589, Saudi Arabia; 3 Department of Pharmaceutics and Pharmacokinetics, CSIR-Central Drug Research Institute, Lucknow 226031, India; 4 Department of Biology, College of Science and Arts at Alkamil, University of Jeddah, Jeddah 23218, Saudi Arabia; 5 Department of Kuliyate Tib, National Institute of Unani Medicine, Kottigepalya, Bengaluru 560091, India; 6 Department of Medical Laboratory Sciences, Faculty of Applied Medical Sciences, King Abdulaziz University, Jeddah 21589, Saudi Arabia; 7 Vaccines and Immunotherapy Unit, King Fahd Medical Research Center, King Abdulaziz University, Jeddah 21589, Saudi Arabia; 8 King Fahd Medical Research Center, King Abdulaziz University, Jeddah 21589, Saudi Arabia

**Keywords:** brain metastases, breast cancer, chemokine receptor, HER2, TNBC

## Abstract

Brain metastasis is one of the major reasons of death in breast cancer (BC) patients, significantly affecting the quality of life, physical activity, and interdependence on several individuals. There is no clear evidence in scientific literature that depicts an exact mechanism relating to brain metastasis in BC patients. The tendency to develop breast cancer brain metastases (BCBMs) differs by the BC subtype, varying from almost half with triple-negative breast cancer (TNBC) (HER2^−^ ER^−^ PR^−^), one-third with HER2^+^ (human epidermal growth factor receptor 2-positive, and around one-tenth with luminal subclass (ER^+^ (estrogen positive) or PR^+^ (progesterone positive)) breast cancer. This review focuses on the molecular pathways as possible therapeutic targets of BCBMs and their potent drugs under different stages of clinical trial. In view of increased numbers of clinical trials and systemic studies, the scientific community is hopeful of unraveling the underlying mechanisms of BCBMs that will help in designing an effective treatment regimen with multiple molecular targets.

## 1. Introduction

Cancer has a common characteristic of dysregulated cell growth composed of a heterogeneous group of ailments [1,2]. It evolved from an amalgamation of genetic and epigenetic aberrations that cause the switch-off/on of tumor suppressor genes (TSGs) and oncogenes [2,3]. Among different cancer types, breast cancer (BC) is a heterogeneous ailment that consists of specific biological sub-divisions [4]. Nearly half a million people die annually due to BC worldwide. The global incidence of cancer has increased to 19.3 million newly identified cases and 10 million cancer-associated mortalities per year [5,6,7]. BC is the most common type of cancer worldwide and the fifth leading cause of cancer-associated deaths amongst females, with an estimated incidence of 2.3 million (11.7%) new cases and 684,996 (6.9%) deaths in 2020 [7].

BC is further classified into several subclasses, characterized by immunohistochemical staining {(e.g., ER, PR, HER2 (ERBB2)}, proliferation marker protein Ki-67 (MKI67), genomic markers (e.g., BRCA1, BRCA2, and PIK3CA), and immunomarkers (e.g., tumor-infiltrating lymphocytes and PD-L1) [8]. Conventionally, the treatment of BC is based on the expression of the progesterone receptor (PR), estrogen receptor (ER), and human epidermal growth factor receptor 2 (HER2). The presence of these biomarkers has facilitated the development of effective and targeted treatments. Moreover, chemotherapy is the only therapeutic option for tumors with triple-negative breast cancer (TNBC), which lacks expression of ER, PR, and overexpression of HER2 [2,9,10]. 

One of the deadliest diseases that affect people is brain tumors, which have a high relapse rate and only a moderate to poor prognosis. It can be divided into two main categories: primary brain tumors that begin in the brain and secondary brain tumors that are produced by cancer cells that have spread from tumors and are established in other regions of the body. Primary brain tumors can originate from various types of brain cells, the meninges, and nerves. Gliomas, which develop from the brain’s glial tissue, are the most characteristic primary tumor type [11].

The blood-brain barrier (BBB) is one of the major challenging factors in treating brain tumors [12]. The BBB comprises various molecular parts and transport mechanisms, which produce machinery for drug efflux or obstructions to drug entry into the brain. Therefore, to tackle such types of challenges in treating brain tumors, efficient drug delivery alteration and innovative therapeutic approaches are required, in addition to traditional strategies [12].

Secondary brain tumors or brain metastases cause barrier degradation, culminating in a significant rise in membrane permeability throughout and outside the tumor mass [12]. The aggregation of circulating tumor cells within the brain microvasculature largely initiates brain metastases. The distinct microenvironment of brain cells further restricts the entry of systemic therapies and promotes tumor growth. Due to BBB breakdown, the highly proliferating metastatic cancerous cell enters the CNS mainly through the bloodstream [13]. These tumor cells then rapidly divide and cause local invasion, inflammation, displacement, and edema. The cancerous cells are concentrated in the brain areas with a greater blood flow supply, while the distribution of tumor cells among different brain regions varies with the histological subtypes [14,15].

## 2. Brain Metastases (BMs)

The most common malignant tumors of the central nervous system (CNS) are brain metastases [16]. Brain metastases (BMs) are ten times more prevalent than primary brain tumors, affecting 10% to 20% of adult cancer patients [17]. The most frequent causes of BMs are lung adenocarcinoma, breast carcinoma, and melanoma [18]. Brain metastasis in breast cancer is frequently identified in patients with the advanced-stage illness. It has a poor prognosis because the blood-brain barrier (BBB) hinders the delivery of various medications in the CNS [19]. Some leading causes of brain metastases result in renal cell carcinoma (melanoma), breast, colorectal, and lung cancer, which are associated with poor survival [20]. The metastatic property of breast carcinoma cells is not restricted to the brain. It could affect several other organs, too, such as the liver, distant lymph nodes, lungs, and bones [21]. Approximately 10–30% of women with metastatic breast cancer develop BMs [21]. However, people with subtypes of breast cancer commonly experience metastasis of cancer cells in the brain. Patients with HER2-positive or triple-negative breast cancer are at a higher risk of developing brain metastases [21].

The incidence of brain metastases seems to have increased in recent years, likely due to the prolonged survival of patients receiving effective treatments and the availability of better imaging techniques that lead to improved detection of brain metastases [19,22]. Table 1 summarizes different risk factors, their identification, and chances of BM in BC patients.

## 3. Breast Cancer Brain Metastases (BCBM)

Breast cancer brain metastases (BCBM) are the second most frequent type of brain metastases and one of the most typical breast cancer metastases [31,32]. Scientific studies have identified that patients with breast cancer had a 5.1% rate of BCBM incidence. Moreover, among patients with any metastatic disease, 14.2% developed BCBM during the therapeutic phase of the illness [32]. Most BM occurs in patients with HER2-positive and ER-negative metastatic BC. Among these, HER2-positive BC patients have a higher rate of survival. Due to the therapeutic challenges, BCBM requires an integrated therapy approach for its management [33].

**Figure 1:**
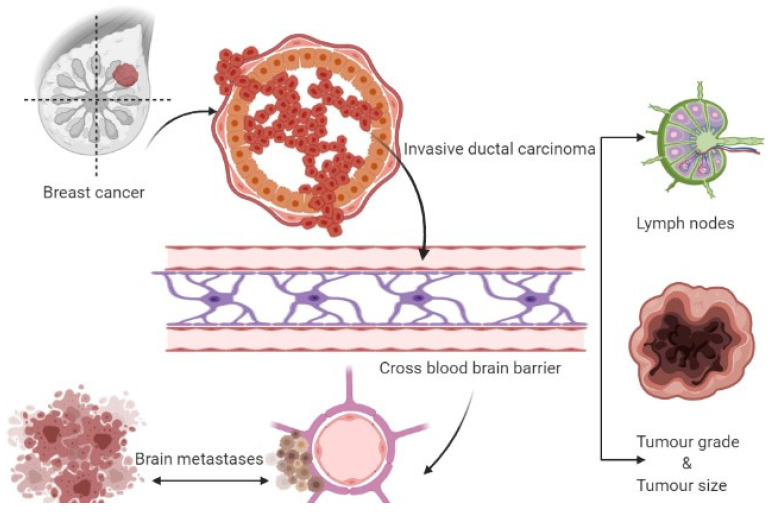
Formation of breast cancer-associated brain tumors.

Three therapeutic approaches are available for treating metastatic brain tumors, including anticancer agents, surgery, and radiation therapy [19,21,32]. Metastasis represents the primary cause of death in BC patients suffering from BMs, which gradually progresses into a more advanced stage [34]. Metastasized breast cancers or circulating tumor cells evade the BBB, and upon reaching the specified cranial cells (astrocytes), they initiate tumorigenesis leading to tumor formation. The initial growth of BMs in the brain is associated with the entry of cancerous cells into the bloodstream and different locations of the brain, where they grow and multiply rapidly [21]. Hence, further insight into breast cancer brain metastasis mechanisms is expected to provide a mode of management or inhibition of these cancer types. Figure 1 depicts the formation of breast cancer brain tumors.

## 4. Challenges of BM

Despite several available multimodal treatments and more advanced systemic therapies, conventional treatments of BM include surgery, radiotherapy, chemotherapy, and immunotherapy [17]. The rapid development of BMs contributes significantly to overall cancer mortality, mainly in the advanced stage, and is also linked to poor prognosis. Several factors, such as subtype, are a strong prognostic factor for the long-term survival of BC with a high grade of BM [35]. The patient’s survival rate is very low (approx. 3 to 5 months) in the case of the triple-negative cancer grade type. Metastasis of aggressive TNBC has higher apparent diffusion coefficient values than the less aggressive hormone-positive group. An increase in apparent diffusion coefficient values indicates a poor prognosis in patients with brain metastases due to BC [36].

Patients’ age also affects the progression rate, and age > 45 is related to a smaller survival from the first tumor relapse time [23] (American Cancer Society, 2019). In addition, the presence of some extracranial disease leads to the worst case of BM progression in breast cancer patients, representing the overall burden of brain metastases. Hence, tumor grade, and prognostic factor, is an important tool for evaluating progression rate and is defined as graded prognostic assessment (GPA) [37]. Karnofsky’s performance status score, extracranial metastases, and GPA include age and number of tumors in BC brain metastases [32,38]. Recently, surgery and radiotherapy have been the two commonly used treatment methods for metastatic brain cancers [32]. Other systemic methods are still in the developmental stage and are restricted to regulating extracranial malady. However, newer imaging techniques (e.g., response assessment in neuro-oncology brain metastasis) must be employed for evaluation consistency [39]. Diffusion tensor imaging (DTI) plays a crucial role in setting up a suitable treatment methodology for low diffusion coefficient brain metastases due to BC.

## 5. The Blood-Brain Barrier (BBB) and Blood-Tumor Barrier (BTB): A Physiological Barrier in Brain Cancer Treatment

The BBB maintains the CNS structure and function by creating an organized neurovascular unit (NVU), which contains endothelial cells (ECs), pericytes, and astrocytic end feet [40,41]. These structures also prevent the pathway of the drug into brain tumors. The BTB is formed as a result of tumor development and BBB interruption. Scientific studies reported that suboptimal therapies in brain tumors are due to the lesser permeability of BTB than BBB to small and large molecules [42,43].

The BBB has a special structure surrounded by the basal lamina that contains glycoproteins cleaved to perform different functions [44]. In addition, multiple ligands also contribute to its function through distinct signaling cells, viz. ECs, pericytes, and astrocytes [45]. ATP-binding cassette transporters’ (ABC transporters) polarization can regulate the active transport through the BBB [46]. Astrocytes are the most abundant cell type in the brain, and their metabolic sensors play an integral part in BBB development and function [47]. The astrocytic end feet dislocation from an endothelial cell by a single invading tumor cell causes local BBB breaching [48]. Astrocytes and pericytes play important roles in BBB function during development, adulthood, and disease progression. Vascular functions, such as vessel remodeling and neuroinflammation, are regulated by pericytes, sited at the abluminal side of the endothelium. Similarly, the microglia, an innate immune cell, can influence CNS vasculogenesis development and contribute to BBB function [49,50]. GABA-ergic, cholinergic, noradrenergic, and serotonergic neurons can affect the CNS endothelium to maintain blood flow, neurovascular coupling, and BBB permeability at synaptic endings [51].

Some diseases, such as brain cancer, stroke, and autoimmune deficiency syndrome, can modulate the structure and function of BBB [52,53]. The alterations in the BBB are not correlated with tumor size, type, or anatomic location [54]. The endothelial cell-specific mitogen, known as vascular endothelial growth factor (VEGF), is a potent inducer of vascular permeability. VEGF may mediate endothelial cell proliferation and vascular permeability in glial tumors. This association has significance for therapeutic applications, including evaluation of the administration of water-soluble medications, edema treatment, and anti-angiogenesis therapy [55,56]. Compared to the surrounding normal brain, where the BBB is typically intact, high-grade gliomas have a disturbed BTB that allows enhanced drug delivery of the drugs to the tumor core [12].

**Figure 2:**
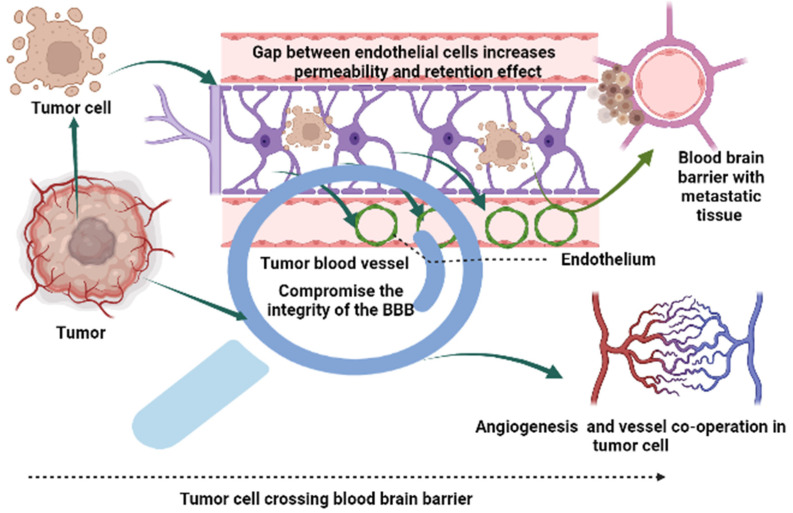
An illustrative image depicting the blood-brain barrier and blood-tumor barrier.

The BTB comprises three distinct micro-vessel accumulations, i.e., continuous, and non-fenestrated capillaries, continuous and fenestrated capillaries, and capillaries containing inter-endothelial gaps [57]. Drug efflux transporters are found in both BBB and BTB endothelial cells [58]. The advanced/invasive glioma cells are spread outside areas of disrupted BTB and inside normal brain regions [59]. In low- and high-grade gliomas, the BBB and BTB barriers thwart the delivery of sufficient therapies. The BTB is formed by brain tumor capillaries and comprises a barrier different from the BBB. The hypoxic areas resulting from metabolic demands of developed glioma lead to high expression of VEGF and angiogenesis, resulting in irregular vessel formation and disruption of BTB function [60,61]. The overexpression of receptors on brain tumor capillaries could be exploited to enhance drug delivery to tumor tissues [62,63]. An illustrative image depicting the BBB/BTB affecting brain cancer treatment has been provided in the Figure 2.

## 6. Treatment Modalities and Challenges

Highly chemotherapy-sensitive primary tumors are only eligible for systemic treatments of BMs. Systemic therapies are recommended in cases where therapeutic options have been exhausted [64]. Hence, the efficacious treatment for BCBMs is still lacking.

## 7. Complete Systemic Targeted Therapy

Systemic targeted therapy designed for BCBM patients is chemotherapy-based cytotoxicity [65]. To obtain a better outcome from targeted therapy, it is crucial to consider the tumor subtype and its prognosis rate.

## 8. Local Therapy Modalities

Patients with a solitary or small number of lesions should preferably go for a surgical line of handling and resection of the BM, specifically, at the time the brain syndrome is fine and the BM is showing symptoms. Except for the limitation of the anatomic location of the metastatic lesion, surgical resection has capability for instant development of intracranial hypertension and establishes histological analysis for patients in which there is no other site of metastasis [23]. In a learning study, 50 patients’ performance at the beginning of BM were studied. Several major tumors were randomized in which additional surgical resection of BMs followed via whole-brain radiation targeting (WBRT), or needle biopsy following WBRT [66]. The recurrence of BMs is less general within the surgical practice set than the emission collection [67]. Meanwhile, surgical procedures or radiotherapy are considered common in the current management plan of BCBMs.

## 9. Surgical Approaches

Systemic medical therapy significantly enhances the survival rate of stage 4 de novo BC patients, as evident from the literature [68,69]. It also shows the effectiveness of primary tumor surgery for a better prognosis at an early stage under specific clinical conditions. On the other hand, multiple surgical approaches provide a better opportunity to reduce the anesthesiologic conditions and unnecessary surgical trauma [70].

## 10. Radiation Targeted Therapy

Whole-brain radiation targeted treatment is an extremely vital line of a BC cure, especially for multiple targeted lesions in the brain. The two major goals of WBRT are the eradication of microscopic seeding of the brain and controlling macroscopic metastases [66]. The benefit of this therapy was well received in 95 randomized trials with various age groups of patients with BMBC. It was observed that the radiation-treated group had a small recurrence at the operation site and in some other areas of the brain, however, the complete survival rate was not increased [71].

## 11. Stereotactic Radiotherapy

**Figure 3:**
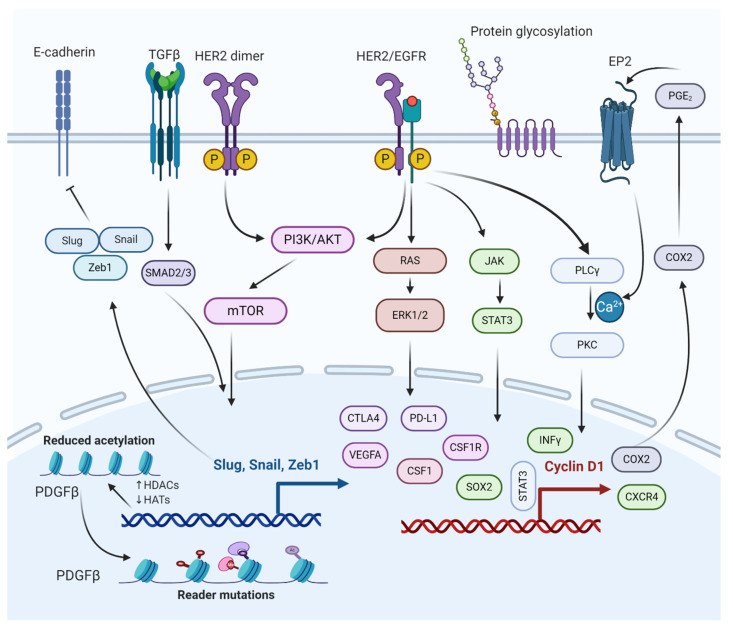
Molecular interactions of different pathways involved in the regulation of BCBM. The epithelial to mesenchymal transition (EMT) genes inhibit cadherin, promoting metastasis. PI3K/AKT/mTOR and RAS/RAF/ERK pathways activate cellular processes, such as cell proliferation, survival, migration, and angiogenesis. PI3K/AKT/mTOR and JAK/STAT pathways help BCBMs escape immunosurveillance. COX2 aids in prostaglandin synthesis and inflammation activation. Histone deacetylation of growth factors and protein glycosylation aid in bypassing the BBB.

This has been suggested as a substitute management option for patients with some degree of BM or lesion at difficult anatomic locations [72]. This method provides high-precision photon radiation in smaller volumes targeting and carefully avoiding the major parts of brain tissues. WBRT causes toxicity leading to neurocognitive decline, while stereotactic radiotherapy does not show such effect [35]. In the below-mentioned sections, we have covered different molecular pathways involved in regulating BCBM. Because of their significant role in BCBM, these pathways could be potential therapeutic targets for BCBM treatment. The molecular interactions of different pathways involved in BCBM have been depicted in Figure 3.

## 12. Molecular Pathways Involved in the Regulation of BCBM

### 12.1. TGFβ/SMAD Signaling Pathway

It is well-known that epithelial to mesenchymal transition (EMT) proteins such as Slug, Snail, and Zeb1 are expressed through transforming growth factor β (TGFβ), SMAD, and PI3K signaling pathways (Figure 3) [73]. The EMT proteins promote this transition by decreasing the expression of E-cadherin and increasing the expression of N-cadherin [74]. EMT plays a significant role in cancer by increasing invasiveness and metastasis, resulting in poor prognosis and survival [75]. The EMT proteins suppress the expression of CDH1 [76]. The CDH1 gene translates to E-cadherin, which plays a critical role in cell adhesion and is involved in cell attachment to other cells and the extracellular matrix (ECM) [77]. Without E-cadherin, the breast cancer cells are detached from the breast tissue, forming circulating tumor cells (CTCs) that can metastasize to other tissues, including the lungs and brain [24]. Under normal physiological conditions, the BBB selectively regulates materials that go into the brain compartment by preventing the paracellular diffusion of compounds. This causes an obstacle for breast CTCs to pass through the BBB; however, in BCBM, the breast CTCs diffuse through the endothelial cell junctions [24,78]. The endothelial cell junctions are the part of the BBB that is modified during BM formation. Slug, Snail, Zeb1, VEGFA, and CD44 contribute to BM formation by enhancing the trans-endothelial migration of tumor cells via downregulation of endothelial integrity, enabling the breast CTCs to pass the BBB [79] (Mittal, 2018). Targeting the SMAD protein in the TGFβ/SMAD signaling pathway has been suggested to attenuate brain metastasis in BCBM patients [80]. The lipoprotein receptor-related protein 1 (LRP-1) inhibitors (ANG1005 and GRN1005) bind to LRP-1, leading to LRP-1 receptor-mediated transcytosis or endocytosis across the BBB, resulting in tumor growth arrest and apoptosis [81,82].

### 12.2. PI3K/mTOR Signaling Pathway

The phosphoinositide 3-kinase (PI3K) signaling pathway is a central pathway involved in cellular processes such as cell survival, cell proliferation, cell metabolism, and angiogenesis (Figure 3) [83,84,85]. It also plays a significant role in BCBM with approximately 77% of patients having been noted to have an activated PI3K signaling pathway [86,87]. The activation of the PI3K signaling pathway is associated with increased expression of metastatic and immunosuppressive genes, which include CTLA4, PD-L1, CSF1R, and CSF1 in the tumor microenvironment of metastasized brain cells [21,88]. The loss of function of phosphatase and tensin homolog (PTEN), a tumor suppressor and a negative regulator of PI3K signaling, is detected in 25–71% of BCBM patients, with the highest percentage in TNBC cases [89]. Overexpression of PTEN in astrocytes suppresses invasiveness and cell migration, suggesting PTEN as a promising therapeutic target for BCBM treatment [87]. The mTOR is a serine/threonine protein kinase, a downstream protein of PI3K, and Akt plays a significant role in several cancer types [84,90]. Simultaneous mTOR and PI3K protein inhibition have been reported to attenuate BCBM [91]. Everolimus and Buparlisib (BKM120), mTOR and PI3K inhibitors, are used to treat BCBM in combination with other anticancer drugs such as trastuzumab and vinorelbine [38].

### 12.3. HER2/Epidermal Growth Factor Receptor (EGFR) Signaling Pathway

EGFR is a transmembrane protein that activates the EGFR signaling pathway through homo/hetero-dimerization and auto-phosphorylation in response to ligand binding [92]. EGFR forms heterodimer with HER2, activating the PI3K/AKT signaling cascade. HER2^+^ breast cancer is susceptible to brain metastasis due to its link with PI3K signaling pathway (Figure 3) [93]. The HER2 protein dimerizes with another similar protein called HER3, triggering cell proliferation and survival. One study based on immune-histochemistry reported that HER3 is over-expressed in around 60% of BCBM patients [94].

HER2 signaling is a master regulator of many pro-inflammatory, proliferative, and pro-metastatic pathways, the most notable of which is the cyclo-oxygenase 2 (COX2) [95,96]. The HER2/EGFR signaling pathway is directly or indirectly associated with COX2 upregulation, which has shown to induce specified brain metastasis. Because BCBM patients have high expression of both HER2 and EGFR, several drugs (some approved and others in clinical trials) are used to target various stages of the HER2/EGFR signaling pathway [97,98]. Some of the drugs that target HER2/EGFR are Lapatinib (targets HER2 receptor), Trastuzumab (targets HER2 receptor), KD019 (targets HER2, Src, and EGFR), ARRY-380 (targets HER2 receptor), HKI-272 (targets HER1, HER2, and HER4 receptors), Afatinib (targets EGFR1, EGFR2, and EGFR4 receptors), and tucatinib (targets HER2 receptor). It is worth mentioning that tucatinib, a tyrosine kinase inhibitor, combined with trastuzumab and capecitabine, was approved by the USFDA on April 17, 2020, as a BCBM treatment regimen [38,99,100].

### 12.4. JAK/Signal Transducer and Activator of Transcription 3 (STAT3) Signaling Pathway

The JAK-STAT pathway is known for regulating the expression of growth factors and cytokines [101]. Some of the genes involved in the JAK-STAT pathway include PD-L1, VEGFA, and CTLA4, which play a crucial role in the survival of BCBM by escaping from immunosurveillance (Figure 3) [102]. STAT3 is critical for astrocytic scar formation and is involved in axon regeneration [103] (Anderson et al., 2016). Most astrocytes in BMs are expressed as an activated form of STAT3, the phosphorylated STAT3 (pSTAT3) [104] (Priego et al., 2018). The pSTAT3^+^ cancerous astrocytes bypass immunosurveillance by expressing escape-promoting genes, such as PD-L1, CTLA4, VEGFA, and TIMP-1 [105]. Therefore, STAT3 in BMs could be a potential therapeutic target for BCBM treatment. Nivolumab, an approved anticancer drug, targets PD-1 in BCBM, thereby preventing the binding of PD-L1 to PD-1. Similarly, using nivolumab and other treatment regimens helps cancer immunotherapy [106,107].

## 13. Role of Oncogenes Regulating Brain Metastasis through Breast Cancer Cells Growth Inhibition

### 13.1. Role of CXCR4 Gene

Gene therapy signifies a potential method for the treatment of BCBM. A nano-sized poly(lactone-co-β-amino ester) has been reported to deliver the CXC chemokine receptor 4 (CXCR4) gene to target BCBM via surface conjugation of AMD3100 in the cancer microenvironment [108]. Promelittin (proMel) is delineated to manifest secretory protein and discharges cytolytic melittin due to MMP-2 stimulation gathered at tumor sites. The release of proMel efficiently impedes cancer development through the AMD3100-conjugated nanoparticles in a BCBM mouse model. This is an innovative strategy for treating BCBM via targeted delivery of promelittin-led gene therapy [109].

### 13.2. Role of SOX2 Gene

SOX2 is overexpressed in cancer cells, helping in the adhesion of cancerous cells to the endothelial (microvascular) cells. It promotes trans-endothelial migration and BBB permeability, while the silencing of SOX2 prevents these incidents. These functions of SOX2 are possibly due to the upregulation of HBEGF and FSCN1, involving AKT and β-catenin signaling pathways. A recent in vivo study reported that SOX2 enhances the growth of BCBM [110].

### 13.3. Role of BRCA1/2 Gene

Approximately 5-10% of breast cancer cases are associated with the hereditary mutation of BRCA1 and BRCA1 genes [111]. The induction of BMs is common among females with BRCA2/BRCA1 mutations, especially those suffering from metastatic BC. Moreover, carrier-mediated mutation at BRCA2 shows a considerable rate of recurrence of CNS metastasis than the non-carrier type [112].

The crucial process of single-strand break repair in DNA is absent in BRCA1 and BRCA2 mutations, dramatically increasing breast/ovarian cancer chances. An early study reported a higher rate of BCBM in patients with BRCA1 mutations [113]. Although no statistical correlation was noted between BRCA1 mutation carriers and non-carriers (*p* = 0.06), BRCA1 mutations (58% vs. 24%) are believed to be a common mutation in BCBM patients [114]. A recent study reported that patients with BRCA1 and BRCA2 mutations have significantly higher rates of CNS metastases than non-carriers (BRCA1: 53% and BRCA2: 50% vs. non-carriers: 25%, respectively) [112].

## 14. Emerging Trends of Therapeutics to Treat BMs

Neratinib is an irreversible chloroanilino-quinazoline-derived inhibitor of HER2 [115]. Neratinib, combined with capecitabine, has shown its effectiveness, mainly for managing and treating HER2^+^ metastatic breast cancer (MBC) patients. On the other hand, pyrotinib is more effective and promising against HER2^+^ MBC, a pan-ErbB receptor TKI (tyrosine kinase inhibitor), binds irreversibly, and is orally given [116,117,118,119]. This drug showed encouraging results in intracranial control [93].

## 15. Forecast for BCBM Cure

Bevacizumab, a drug for cancer chemotherapy, has been reported to be effective in glioblastomas and has the potential to block VEGF-like growth factors, which is vital for the cure of BCBM [120]. Two studies supported the above observation. The first study observed 65% CNS responses for etoposide, cisplatin, and bevacizumab, and the second study noted 61% responses for the same drug [121]. However, based on the overall survival rate of brain metastatic cancer patients taking these drugs, it was withdrawn from Food and Drug Administration approval.

Capecitabine along with lapatinib and everolimus, in addition to one more combination of vinorelbine with trastuzumab and everolimus, were evaluated in HER2-positive BCBM patients [122,123]. This study showed a high level of brain metastases in triple-negative BC and HER2-positive patients, highlighting the requirement of cost-effective strategies against brain metastases risk groups and their subgroups [124]. One study reported that the expansion time of BCBM patients through triple-negative and HER2-positive is not comparable [125].

Pertuzumab is regarded as a systemic therapy for BCBM patients, which is a humanized monoclonal antibody that prevents HER2 dimerization ([126]. In patients with HER2^+^ brain metastases, a phase II clinical trial (NCT02536339) is underway to measure the efficacy and safety of a combination of pertuzumab and high doses of trastuzumab. Preliminary data of this finding were published by Lin et al. (2017).

**Figure 4:**
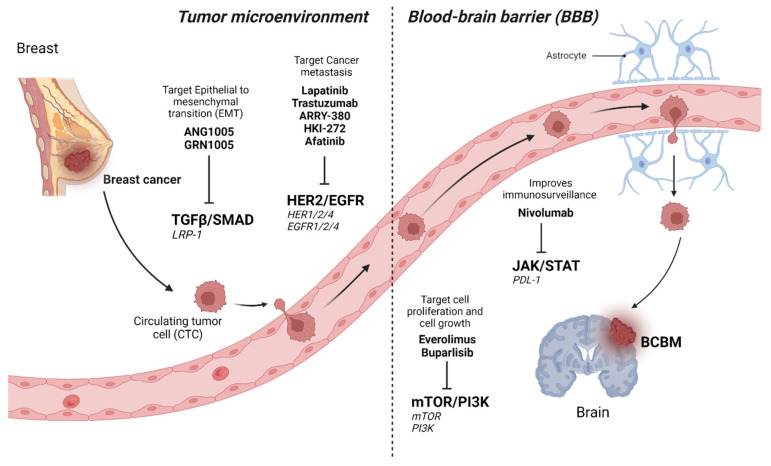
Several signaling cascades involved in BCBM therapeutic targets and their potential drugs.

A monoclonal antibody, a trastuzumab emtansine (T-DM1), is an antibody-drug conjugate made up of trastuzumab, associated with DM1 (a cytotoxic substance maytansine derivative) [127]. Some researchers reported beneficial case studies that the administration of T-DM1 was a well-tolerated therapeutic approach for patients with HER2^+^ BCBM [127,128]. Trastuzumab deruxtecan is an antibody-drug combination containing trastuzumab and a derivative of exatecan (topoisomerase I inhibitor), used for treating BCBM. For HER2^+^ metastatic breast cancer (MBC) that had received T-DM1 treatment previously, the phase II trial DESTINY-Breast01 examined the effectiveness and safety of this combination (NCT03248492) [129,130,131]. Several signaling cascades involved in BCBM, therapeutic targets, and their potential drugs have been depicted in Figure 4.

Tucatinib is a tyrosine kinase inhibitor, highly selective for the HER2 protein and can slightly inhibit the epidermal growth factor receptor [132]. Trastuzumab and capecitabine, in combination with tucatinib, demonstrated improved progression-free survival and enhanced CNS response in the BCBM treatment regimen [38,99,100,132]. Some recent studies also reported mTOR and PI3K inhibitors (Everolimus and Buparlisib (BKM120)) for the treatment of BCBM in combination with other anticancer drugs, such as trastuzumab and vinorelbine [38,133]. Pembrolizumab is a humanized monoclonal IgG4-antibody with high affinity and great selectivity against PD-1 [134]. PD-L1 inhibitors have been evaluated as targeted therapies for advanced BC in various trials. Pembrolizumab exhibited sufficient activity and safety for advanced TNBC, as shown by the KEYNOTE-012 trial (NCT01848834) [32,134]. Stereotactic radiosurgery (SRS) and atezolizumab are combined to manage TNB with BCBM in the phase II atezolizumab trial (NCT03483012) [135]. In addition, pembrolizumab and SRS are being tested in phase I/II trials for patients with BCBM to determine their effectiveness and safety (NCT03449238). In another clinical trial, patients with BCBM are assessed for SRS following nivolumab in phase I (NCT03807765) research [32]. Table 2 summarizes current/clinical trial drugs and their mechanism of action against BCBM.

## 16. Conclusions

Brain metastasis (BM) is common in breast cancer patients with poor prognosis and remains a life-threatening disease. There is a pressing need for effective therapy to cure breast cancers and breast cancer brain metastases. A thorough and detailed understanding of the CNS sequence process requires robust analytical data to manage primary and secondary BM. As the biology of BM is delicate, the challenges associated with BBB need to be handled to treat BCBMs effectively. Moreover, there is a greater risk of BC treatment at the advanced stage due to the more complex tumor microenvironment and deregulatory pathways, ultimately leading to BM. Targeting the mTOR pathway is one of the effective and commonly used treatments for BC patients. However, there is no well-defined treatment plan for brain metastases. Presently, several studies on BCBM are underway, which are expected to help scientists to chalk out an effective treatment strategy against this life threatening disease. In addition, there is an urgent need for multidimensional in vitro and in vivo studies involving the epigenetic players so that the cancer cells can be arrested in the G2/M phase. Furthermore, downregulation of the oncogenes (EGFR, VEGFR, PDGFR, HER2) accompanied by upregulation of the tumor suppressor genes (p53, p73, p21, RB, BRCA1/BRCA2, PTEN gene) can also orchestrate effective apoptosis in a tumor cell.

## Figures and Tables

**Table 1:** Risk factors, identification, and chances of BM in BC patients.

Risk Factors	Identification	Brain Metastasis Risk	References
Lymph nodes	Histopathology	Positive lymphnodes-4, HR = 2.5, *p* = 0.029	[23]
Tumor grade	Histopathology	The grade of the tumor is 3; Rate is 7.9% after 10-years completion of follow-up	[24]
Tumor size	Histopathology	The size of the tumor is 2 cm, after 10 years with a 7% rate. Size of tumor greater than 2 cm have a high risk of BM	[25]
Luminal A and B subtypes	Molecular biology	Over-expresses of epidermal growth factor of a human determined as HER-2	[26]
AlphaB-crystallin (CRYAB)	Genetic biomarker	Occurrence of BM	[27]
Phosphatidylinositol 3-kinase (PI3K) and mitogen-activated protein kinase (MAPK/ERK)	Molecular biomarker	Overexpression of HER 3 receptor MAPK signaling pathway preferentially activated in the BM of cancer patients	[23]
VEGF and CXCR-4	Molecular biomarker	Disrupt the BBB with migration in the parenchyma region	[28]
A2,6-sialyltransferase	Genetic driver	Cancer cell extravasations in the course of the BBB	[29]
Adjuvant trastuzumab trials	Targeted therapy for HER2-positive	BM risk was increased with a range of 1.32 to 1.9	[30]

**Table 2:** Current/clinical trial drugs and their mechanism of action against brain metastatic disease in BC.

Target Drug	Treatment Type	Tumor Type	Mode of Action	References
EP versus IC	Chemotherapy	BCBM subgroup	Topoisomerase-I inhibitor-polymer conjugate	[136]
Ang-1005	Chemotherapy	BCBM with leptomeningeal carcinomatosis	Peptide-paclitaxel conjugate	[137]
Abemaciclib	Targeted therapy	HER2-BC	CDK 4 and 6 inhibitor	[138]
Neratinib monotherapy	Targeted therapy	HER2^+^ progression within CNS	Pan-HER inhibitor	[139]
Cabazitaxel + lapatinib	Chemotherapy	HER2^+^ progression	HER2 positive	[140]
Lapatinib + WBRT	Chemotherapy	Effective therapeutic EGFR family target	HER2	[141]
BKM120 + capecitabine	Triple-negative	Capability toward penetration to the BBB	PI3K	[142]
Pertuzumab	Systemic therapy	HER2^+^ BCBM	Monoclonal antibody	[93,126]
Everolimus and Buparlisib		BCBM	mTOR and PI3K inhibitors	[133,38]
Trastuzumab emtansine		HER2^+^ BCBM	Monoclonal antibody	[127,128]
Trastuzumab deruxtecan	Systemic therapy	BCBM HER2^+^ metastatic breast cancer	Topoisomerase I inhibitor	[129,130,131]
Trastuzumab, capecitabine, with tucatinib	Targeted therapy	HER2^+^ BCBM	Tyrosine kinase inhibitor	[99,132]
Pembrolizumab	High affinity and great selectivity	Advance TNBC	Monoclonal IgG4- antibody	[32,134]
Stereotactic radiosurgery (SRS) and atezolizumab		TNB with BCBM		[32]

## Abbreviations

BBB	Blood–brain barrier
BC	Breast cancer
BCBM	Breast cancer brain metastasis
bHER2-ATC	Biparatopic anti-HER2 antibody-tubulysin conjugate
BMs	Brain metastases
BMsBC	Brain metastases breast cancer
BRCA1	Breast cancer type 1
cANGPTL4	Angiopoietin-like 4 fibrinogen-like domain
CNS	Central nervous system
COX-2/MMP1	Cyclooxygenase 2/Matrix metalloproteinase 1
CXCR4	CXC chemokine receptor 4
DTI	Diffusion tensor imaging
ER	Estrogen
GPA	Graded prognostic assessment
HDACs	Histone deacetylases
HER2	Human epidermal growth factor receptor 2
IGF1R	Type 1 insulin-like growth factor receptor
LncRNA	Long noncoding RNA
MBC	Metastatic breast cancer
MEF2	Myocyte enhancer factor 2
miRNAs	MicroRNAs
MMP-2	Matrix metalloproteinase-2
mTOR	Mammalian target of rapamycin
NPNT	Nephronectin
PCI	Prophylactic cranial irradiation
PDGF	Platelet-derived growth factor
PDGFRβ	Platelet-derived growth factor receptor-beta
PD-1	Programmed death receptor 1
PDXs	Patient-derived xenografts
PI3K/AKT	Phosphatidylinositol-3-kinase/Protein kinase B
PR	Progesterone
RRM2	Ribonucleotide reductase M2
SRS	Stereotactic radiosurgery
T-DM1	Trastuzumab emtansine
TGLI1	Truncated glioma-associated oncogene homolog 1
TNBC	Triple-negative breast cancer
VEGF	Vascular endothelial growth factor
WBRT	Whole-brain radiation targeting
